# A prognostic signature based on snoRNA predicts the overall survival of lower-grade glioma patients

**DOI:** 10.3389/fimmu.2023.1138363

**Published:** 2023-11-01

**Authors:** Yi Zhou, Wen Yin, Yirui Kuang, Zhaoping Wu, Haoxuan Huang, Weidong Liu, Xingjun Jiang, Caiping Ren

**Affiliations:** ^1^ Department of Neurosurgery, Xiangya Hospital, Central South University, Changsha, Hunan, China; ^2^ National Clinical Research Center for Geriatric Disorders, Xiangya Hospital, Central South University, Changsha, Hunan, China; ^3^ Cancer Research Institute, School of Basic Medical Science, Central South University, Changsha, China; ^4^ The NHC Key Laboratory of Carcinogenesis and The Key Laboratory of Carcinogenesis and Cancer Invasion of the Chinese Ministry of Education, Central South University, Changsha, Hunan, China

**Keywords:** snoRNA, lower-grade glioma, prognostic signature, overall survival, interaction network

## Abstract

**Introduction:**

Small nucleolar RNAs (snoRNAs) are a group of non-coding RNAs enriched in the nucleus which direct post-transcriptional modifications of rRNAs, snRNAs and other molecules. Recent studies have suggested that snoRNAs have a significant role in tumor oncogenesis and can be served as prognostic markers for predicting the overall survival of tumor patients.

**Methods:**

We screened 122 survival-related snoRNAs from public databases and eventually selected 7 snoRNAs that were most relevant to the prognosis of lower-grade glioma (LGG) patients for the establishment of the 7-snoRNA prognostic signature. Further, we combined clinical characteristics related to the prognosis of glioma patients and the 7-snoRNA prognostic signature to construct a nomogram.

**Results:**

The prognostic model displayed greater predictive power in both validation set and stratification analysis. Results of enrichment analysis revealed that these snoRNAs mainly participated in the post-transcriptional process such as RNA splicing, metabolism and modifications. In addition, 7-snoRNA prognostic signature were positively correlated with immune scores and expression levels of multiple immune checkpoint molecules, which can be used as potential biomarkers for immunotherapy prediction. From the results of bioinformatics analysis, we inferred that SNORD88C has a major role in the development of glioma, and then performed in vitro experiments to validate it. The results revealed that SNORD88C could promote the proliferation, invasion and migration of glioma cells.

**Discussion:**

We established a 7-snoRNA prognostic signature and nomogram that can be applied to evaluate the survival of LGG patients with good sensitivity and specificity. In addition, SNORD88C could promote the proliferation, migration and invasion of glioma cells and is involved in a variety of biological processes related to DNA and RNA.

## 1 Introduction

Glioma is the most prevalent tumor in the central nervous system, with an overall incidence of 4.67-5.73 per 100 000 persons (1). Historically, glioma is a neuroepithelial tumor that originates from glial cells in the central nervous system. Glial cell tumors include astrocytic, oligodendroglial, oligoastrocytic, ependymal and mixed neuronal-glial tumors (2, 3). According to the WHO histological classification of gliomas, grades 2 and 3 gliomas belong to lower-grade gliomas (LGG) (4). In molecular pathology, IDH mutation status, MGMT promoter methylation status and 1p19q co-deletion status are important molecular pathological features of gliomas and have a significant impact on the prognosis of glioma patients (5). Currently, the main treatments for LGG patients are radiotherapy, chemotherapy and surgery, with surgical resection being the main treatment method (6). However, the tumor’s tendency to infiltrate deep into the parenchyma can make it difficult to completely resect the lesion tissue, thus leading to a poor prognosis (7).

In recent years, non-coding RNAs (ncRNAs), especially long non-coding RNAs (lncRNAs) and microRNAs (miRNAs), have been found to play key roles in cellular activity, particularly in relation to cancer. NcRNAs have been identified to act as oncogenic drivers or tumor suppressors in several major tumor types (8). NcRNAs in different tumors can be used as diagnostic or prognostic biomarkers, as well as new targets for targeted therapy (9). Small nucleolar RNAs (snoRNAs) are a family of short ncRNAs with a length of 60-300 nucleotides, which are enriched in the nucleus and guide post-transcriptional modifications of ribosomal and small nuclear RNAs (snRNAs). Based on the characteristic nucleotide motifs and the associated classical partner proteins, snoRNAs are classified into C/D-box and H/ACA-box subfamilies, of which C/D-box snoRNAs are responsible for 2’-O-methylation and H/ACA-box snoRNAs for pseudouridylation of nucleotides on target molecules (10, 11). Previous studies have revealed that snoRNAs are associated with the development of many cancers and are involved in a variety of cancer biological processes including angiogenesis, activation of invasive and metastatic capabilities and maintenance of proliferative signaling (12). Cornelius Pauli et al. found that high expression of SNORD42A plays an important role in the growth and survival of leukemia cells (13). Chunhong Cui et al. demonstrated experimentally that SNORA65 and SNORA7A/7B deletion inhibits the proliferation and tumorigenic capacity of lung cancer cells (14). In gliomas, Xian-Ru Xia et al. confirmed that overexpression of SNORD44 and its host gene GAS activates caspase-dependent apoptosis signaling pathways to promote apoptosis and is accompanied by inhibition of glioma cell invasion, migration and proliferation (15). In addition, through bioinformatics analyses, some researchers have found that the expression of snoRNAs such as SNORD114-17, SNORA36B, U2, U3, and SNORD78 in head and neck squamous carcinoma (16) and SNORA2, SNORD116-2, SNORA59B, SNORD93, SNORD12B, SNORA70B in clear cell renal cell carcinoma (17), correlates with the prognosis of tumor patients, which can be used as prognostic biomarkers for tumor patients.

Despite the continuous increasing of snoRNA researches in cancer, the relationship between snoRNA and LGG is not fully understood; therefore, identifying snoRNAs associated with LGG is urgently warranted and will provide new directions for improving the diagnosis, prognosis and targeted therapy of LGG. Based on the SNORic database which systematically analyzed snoRNA expression across 31 cancer types from The Cancer Genome Atlas (18), we first screened prognostically relevant snoRNAs from survival data and snoRNA expression matrices of 476 LGG patients by using univariate Cox regression analysis. Next, we constructed a 7-snoRNAs prognostic signature using LASSO regression and multivariate Cox regression analyses. Further, we evaluated the clinical predictive efficacy of the 7-snoRNAs prognostic signature and validated it in a test set. Finally, we predicted the biological functions of snoRNAs in the model and experimentally validated the role of SNORD88C in glioma cell lines.

## 2 Materials and methods

### 2.1 TCGA-LGG datasets procurement and preparation

**Figure 1:**
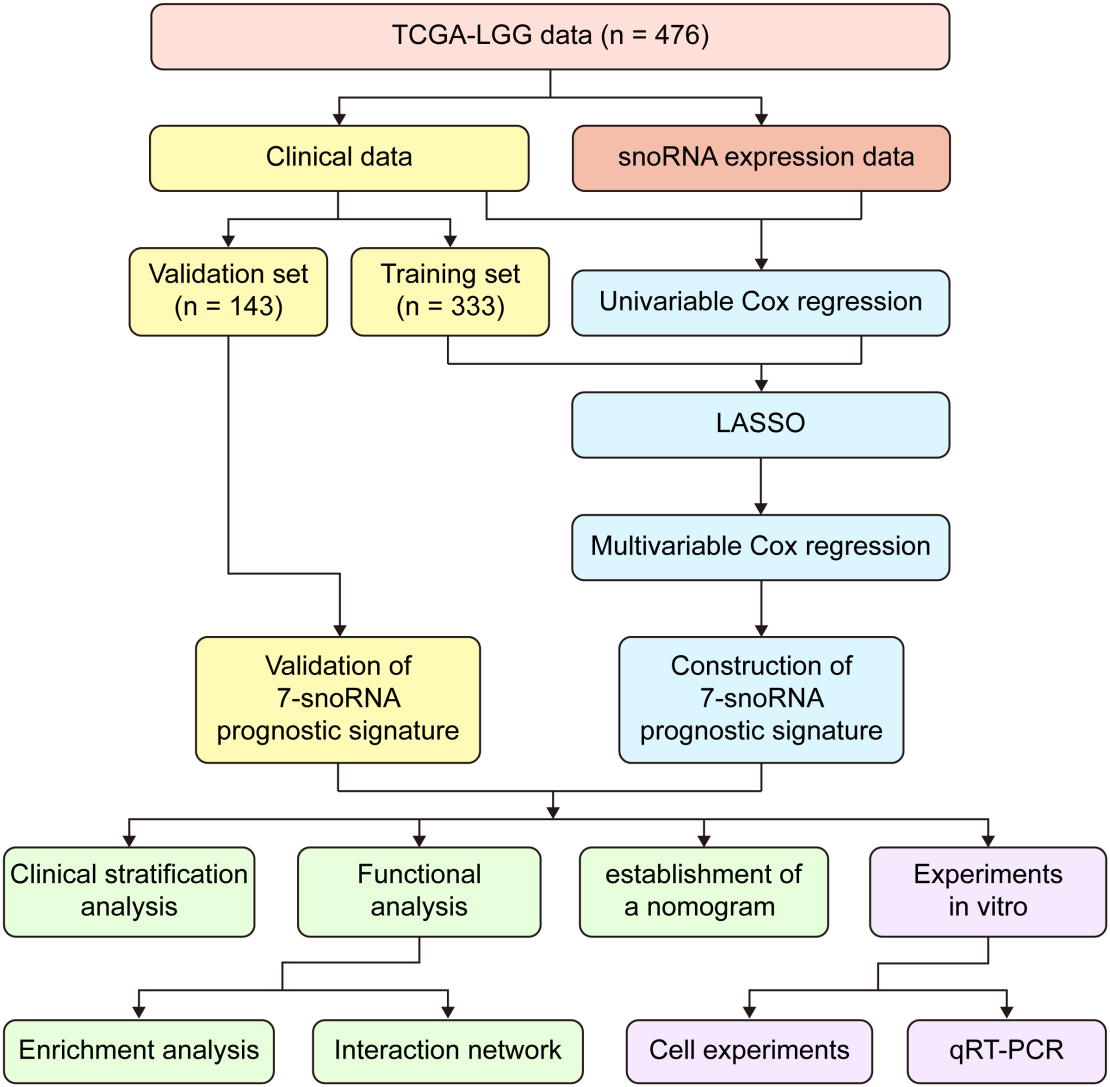
Workflow chart of the entire study.

We downloaded the snoRNA expression matrix of TCGA-LGG patients from the SNORic database(http://bioinfo.life.hust.edu.cn/SNORic/) (18) in the form of Reads Per Kilobase per Million (RPKM) and TCGA-LGG clinical data from database UCSC Xena (https://xenabrowser.net/datapages/). Then, the snoRNA expression data were log (RPKM+1) transformed for the next analysis. To avoid bias in the data analysis, we obtained data for 476 patients (**Table S1**) after removing patients with missing survival data or OS (overall survival) time less than 30 days. Further, we defined that the snoRNAs with no expression in less than 25% of the total number of patients as effectively expressed snoRNAs in LGG. Finally, we selected 476 cases and 358 effectively expressed snoRNAs for the corresponding analysis. Besides, we downloaded the gene expression RNAseq – HTSeq dataset of TCGA-LGG in the form of log2(FPKM+1) from the database UCSC Xena and selected the data corresponding to 476 patients. The workflow chart of this study was illustrated in (**Figure 1**).

### 2.2 Construction of prognostic signature

Data from 476 patients were involved in the construction of the prognostic model. We first randomly divided these patients into a training set (n = 333) and a validation set (n =143) in a ratio of 7:3. Then we used the univariable Cox regression method to identity the survival-related snoRNAs in the whole set (n = 476) with cut-off value *P*< 0.05, and finally got 122 survival-related snoRNAs using R package ‘survival’ version 3.2-10. Subsequently, R package ‘glmnet’ (version 4.1-1) (19) was performed to conduct least absolute shrinkage and selection operator (LASSO) with a ten-fold cross-validation method. LASSO regression was used to select the most prognostically relevant snoRNAs from survival-related snoRNAs and then multivariable Cox regression was performed with cut-off value *P*< 0.05 to screen out the most powerful snoRNAs for the construction of prognostic signature. Ultimately, 7 snoRNAs were identified for model construction. The risk score is calculated by means of 7-snoRNA prognostic signature as follows:


$$Risk\;Score=\;\sum_{i=1}^{n}\beta_{i}\times X_{i}$$

$\beta_{i}$ represented the coefficients of each snoRNA calculated by the multivariable Cox regression and the $X_{i}$ indicated the expression value in the form of log2(RPKM+1).

### 2.3 Construction of nomogram

To plot the nomogram, age, WHO grade and risk scores calculated by 7-snoRNA prognostic signature were screened out by univariate Cox regression for constructing the prognostic model. Then, a multivariate Cox proportional hazard regression model was constructed in the training set. Finally, the nomogram was plotted by using the R package ‘rms’ (version 6.2-0) to predict the probability of survival at 1,3,5 years for LGG patients.

### 2.4 GO term and KEGG pathway analysis

We selected 100 proteins that interacted with each snoRNAs from the RNA Interactome Database (20), followed by Gene Ontology (GO) and Kyoto Encyclopedia of Genes and Genomes (KEGG) enrichment analyses. GO and KEGG analysis was performed by R package ‘clusterProfiler’ (version 3.18.1) (21) and R Package ‘org.Hs.eg.db’ (version 3.12.0), *P*-value< 0.05 was considered statistically significant. The results of GO analysis included Biological Process (BP), Cellular Component (CC) and Molecular Function (MF), each section shows the five most significantly enriched GO terms. KEGG analysis revealed the most 10 significantly enriched pathways.

### 2.5 Gene set enrichment analysis

GESA was performed for the mRNA expression matrix of the high-risk group and low-risk group by software ‘GSEA’ (version 4.1.0). Hallmark gene set (h.all.v7.4.symbols.gmt) was set as reference to identify the different Hallmarks between the high-risk group and low-risk group. |NES|>1, Nom *P*-value< 0.05 and FDR q-value<0.25 were set as screening criteria.

### 2.6 Analysis of the immune characteristics of gliomas

ESTIMATE Score, Immune Score, Stromal Score and tumor purity were calculated by using R package ‘estimate’ (version 1.0.13) (22). In addition, patients were divided into high-immunity group and low-immunity group by the median of ESTIMATE Score. The abundance of 22 types of immune cells was calculated by the CIBERSORT algorithm (23).

### 2.7 Cell culture and quantitative real-time PCR

Glioma cell lines (SHG44 and U251) were obtained from Xiangya Medical school of Central South University, Changsha, China, and were cultured with Dulbecco’s modified Eagle medium (DMEM)-high glucose (Biological Industries, Israel) containing 10% fetal bovine serum (purchased from QmSuero/Tsingmu Biotechnology, Wuhan). Cells were cultured at 37°C in a humidified atmosphere containing 5% carbon dioxide. The siRNA against SNORD88C (target sequences, 5’-GCTCCCATGATGTCCAGCA-3’) was purchased from RiboBio Corporation (Guangzhou, China). Glioma cells were transfected with siRNAs using Lipofectamine 3000 kit (Invitrogen, USA) according to the manufacturer’s direction. The total RNA was extracted using the trizol RNA extraction method and a RevertAid First Strand cDNA Synthesis Kit (Thermo Scientific, Waltham, MA) was used to reversely transcribe RNA to cDNA. Quantitative real time-PCR (qRT-PCR) was used to assess the expression of SNORD88C according to the manufacturer’s instruction (SYBR Green Master Mix, Vazyme). 2^-ΔΔCt^ method was used to calculate relative expression of the gene. Sangon (Shanghai, China) provided the service of synthesizing primers and the sequences are as follows: Human GAPDH forward (5’-CATTGACCTCAACTACATGGTT-3’), reverse (5’-CCATTGATGACAAGCTTCCC-3’); Human SNORD88C forward (5’-CTGATCACCCCTGAGGACACA-3’), reverse (5’-TGTCAAAGGTCCTGGGGTGCA-3’).

### 2.8 Cell proliferation assay

The Cell Counting Kit-8 (CCK-8) assay was used for measuring cell proliferation. Glioma cells were seeded into a 96-well plate (1500 cells/well) after transfecting for 24 hours. At each detection time point, 100ul DMEM containing 10% CCK-8 reagent was added to each well. Then, the absorbance of each well was measured at a wavelength of 450nm after incubation in an incubator at 37°C for 90 minutes.

### 2.9 Cell colony formation assay

Glioma cells were seeded into 6-well plate at a density of 1000 cells per well after transfecting for 24 hours. The culture medium was changed every three days. The cell colonies were fixed with 4% paraformaldehyde for 30 minutes after 10-14 days of culture, subsequently stained with 0.1% crystal violet, and then the number of colonies in each well was counted.

### 2.10 Transwell assays

Transwell assays were conducted by using previously described methods (23).

### 2.11 Clinical sample collection and processing

82 glioma specimens collected by the Department of Neurosurgery of Xiangya Hospital were preserved in liquid nitrogen and formalin, respectively. Formalin-fixed specimens were paraffin-embedded and then made into tissue microarrays. Frozen specimens were extracted for RNA and reverse transcribed to cDNA, and the target snoRNA expression was further detected by qRT-PCR assay (The method is as described previously).

### 2.12 Immunohistochemistry staining

The tissue microarrays were first subjected to antigen repair by boiling antigen repair solution (Servicebio, Wuhan, China), followed by blocking peroxidase activity by 3% hydrogen peroxide (Servicebio, Wuhan, China). CD274 rabbit polyclonal antibody at a dilution of 1:500 (CD274, Cat No: 28076-1-AP, proteintech, China) were incubated overnight at 4°C after being blocked for 1h at room temperature using goat serum. On the next day, the Goat Anti-rabbit IgG/HRP antibody (Cat No: G1213-100UL, Servicebio, Wuhan, China) was incubated for 1h at 37°C and then developed using 3-3’-diaminobenzidine chromogenic solution and finally stained with hematoxylin.

### 2.13 RNA sequencing analysis

U251-NC and U251-si-SNORD88C groups were set up, and the cells were collected after 48h of treatment respectively, and cellular RNA was extracted by using TRIzol. Then RNA quality was analyzed by 5300 Bioanalyser (Agilent) and quantified using the ND-2000 (NanoDrop Technologies). RNA purification, reverse transcription, library construction and sequencing were performed at Shanghai Majorbio Bio-pharm Biotechnology Co.Ltd. (Shanghai, China). The RNA-seq transcriptome library was prepared following Illumina^®^ Stranded mRNA Prep, Ligation from Illumina (San Diego, CA) using 1μg of total RNA. After quantified by Qubit 4.0, paired-end RNA-seq sequencing library was sequenced with the NovaSeq Xplus sequencer. Sequencing data were separately aligned to reference genome with orientation mode and further converted to count and TPM for the following analysis.

### 2.14 Statistics analysis

The Kaplan–Meier survival curve and log-rank test were performed to evaluate the overall survival of patients in the high-risk and low-risk groups. The receiver operating characteristic (ROC) curve was used to evaluate the validity of the prognostic signature, and the area under the curve (AUC) value was calculated to objectively reflect the accuracy of the model prediction ability. The Student’s t-test was used for hypothesis testing of two independent samples that conform to a normal distribution. The Mann-Whitney U test was used for hypothesis testing of two independent samples that do not satisfy the normal distribution. All plots were drawn by the R package ‘ggplot2’ (version 3.3.3) (24) or R basic plotting function.

## 3 Results

### 3.1 Construction of 7-snoRNA prognostic signature in the training set

**Figure 2:**
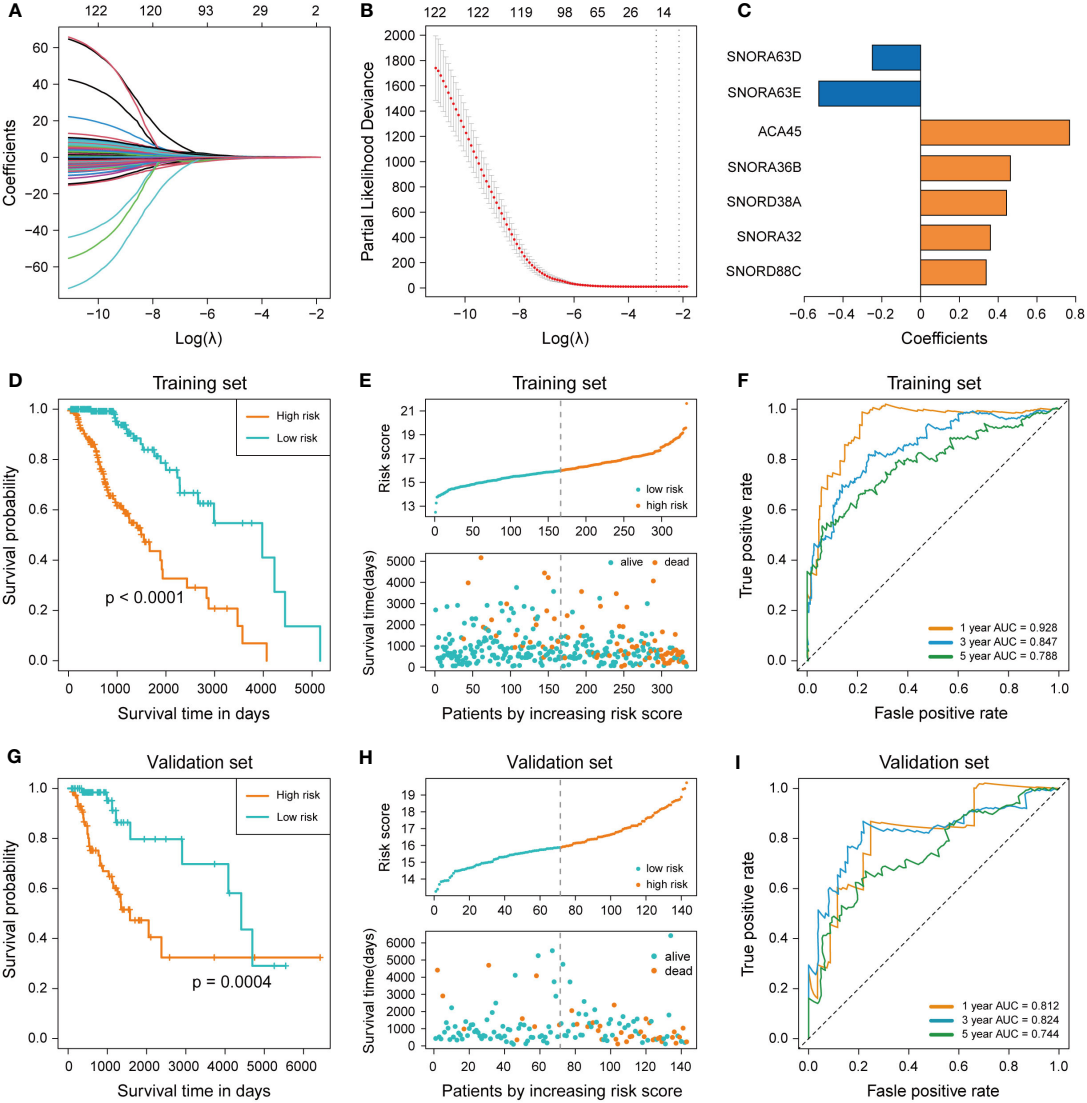
Construction and validation of 7-snoRNA prognostic signature. **(A, B)** The least absolute shrinkage and selection operator (LASSO) regression **(A)** and ten-fold cross-validation **(B)** were performed to calculate the minimum criteria. **(C)** Coefficients of 7 snoRNAs screened out by multivariable Cox regression. **(D, G)** Kaplan–Meier survival curve was plotted to compare the overall survival (OS) between the high-risk group and low-risk group in the training set **(D)** and validation set **(G)**. **(E, H)** The distribution of risk score, survival time and status event in the training set **(E)** and validation set **(H)**. **(F, I)** Time-dependent ROC curve for predicting 1-, 3-, 5-years survival in the training set **(F)** and validation set **(I)**.

In order to construct the prognostic signature, LASSO regression was performed to select the most powerful variable from the 122 survival-related snoRNAs in the training set. Minimum lambda was chosen by the ten-fold cross-validation to seek out the best combination of variables and the outcome was a combination of 15 snoRNAs (**Figures 2A, B**). Multivariable Cox regression analysis was performed for these 15 snoRNAs, and variables with *P*< 0.05 were screened out to enter the regression model. Finally, we constructed a prognostic signature of LGG containing 7 snoRNAs (SNORA32: ENSG00000206799, SNORA36B: ENSG00000222370, SCARNA15: ENSG00000277864, SNORA63E: ENSG00000199363, SNORA63D: ENSG00000201229, SNORD88C: ENSG00000220988, SNORD38A: ENSG00000202031), and the coefficients of each snoRNA are shown in (**Figure 2C**). The Risk Scores (RS) of each patient were calculated by the 7-snoRNA prognostic signature, and the patients in the training set were divided into a high-risk group (RS ≥ median RS) and a low-risk group (RS< median RS) by the median RS. The Kaplan–Meier survival curve showed a significant difference in survival conditions between the high- and low-risk groups, with patients in the high-risk group having a lower survival probability and a shorter overall survival time compared to patients in the low-risk group (**Figure 2D**). In addition, higher RS was associated with shorter survival time and higher mortality (**Figure 2E**). The ROC curves demonstrated the accuracy of the 7-snoRNA prognostic signature in predicting survival at 1,3,5 years in the training set. The AUC values corresponding to 1,3,5 years were 0.928, 0.847, 0.788 respectively, indicating that this prediction model has high specificity and sensitivity (**Figure 2F**).

### 3.2 Validation of 7-snoRNA prognostic signature in the validation set

To verify the ability of the 7-snoRNA prognostic signature to predict the prognosis of LGG patients, we calculated the RS of all patients in the validation set by the prognostic signature and divided them into high- and low-risk groups based on the median RS. Similarly, there was a significant difference in survival between the high and low-risk groups in the validation set, with LGG patients in the high-risk group having a lower survival probability, shorter survival time (**Figure 2G**), and more deaths (**Figure 2H**) compared to the low-risk group, these conclusions were consistent with the results in the training set. The ROC curve of the validation set showed that the AUC for 1, 3, 5 years were 0.812, 0.824, 0.744 (**Figure 2I**). These results suggested that the 7-snoRNA prognostic signature remains a robust predictive model for the prognosis of LGG patients in the validation set.

### 3.3 Effect of snoRNAs on the prognosis of LGG patients

**Figure 3:**
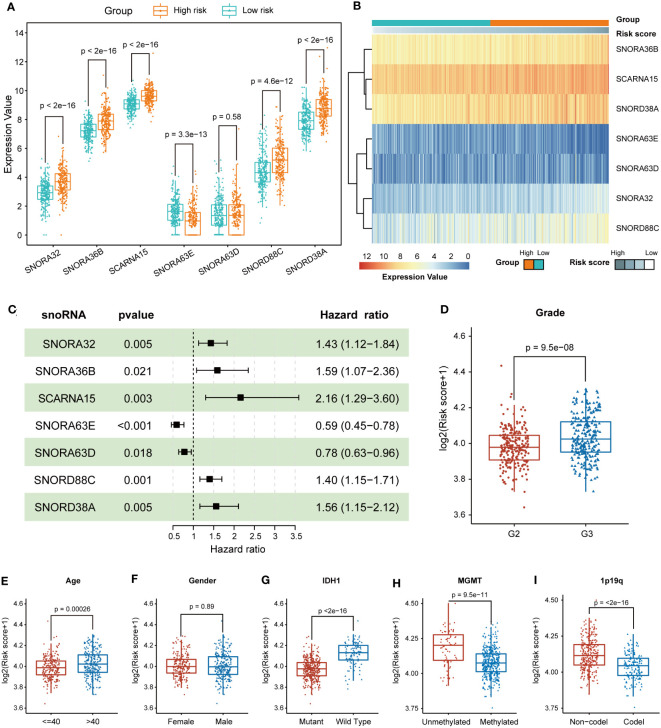
Prognostic and expression features of the 7 snoRNAs. **(A)** Box plots illustrate the expression values of the 7 snoRNAs between the high-risk and low-risk groups **(B)** The heatmap shows the relationship between the expression values of 7 snoRNAs and risk scores. **(C)** The forest plot indicates the multivariable Cox regression results of the 7 snoRNAs included in the prognostic signature. **(D-I)** The differences in risk scores were compared in subgroups, including grade **(D)**, age **(E)**, gender **(F)**, IDH1 mutation status **(G)**, MGMT promoter methylation status **(H)** and 1p19q co-deletion status **(I)**.

To investigate the effects of different snoRNAs on the prognosis of LGG patients, we first compared the differences in snoRNA expression between the low-risk and high-risk groups. SNORA32, SNORA36B, SCARNA15, SNORD88C, and SNORD38A were upregulated in the high-risk group, indicating that they were risky factors in LGG patients. SNORA63E were downregulated in the high-risk group, which means that it may be a protective factor. Nevertheless, the significance of SNORA63D remained unknown, as there was no significant difference in expression between high and low-risk groups (**Figure 3A**). The heatmap revealed that the expression of SNORA36B, SCARNA15, SNORD38A, SNORA32, and SNORD88C increased with increasing risk score, and that of SNORA63E and SNORA63D decreased with increasing risk score (**Figure 3B**). The forest plot showed the results of the multivariable Cox regression of 7-snoRNA prognostic signature. The Hazard ratio (HR) of SNORA32, SNORA36B, SCARNA15, SNORD88C, and SNORD38A were >1, indicating that they were risky factors. SNORA63D and SNORA63E were protective factors in LGG patients with HR<1 (**Figure 3C**).

### 3.4 Stratification analysis to validate the predictive value of the 7-snoRNA prognostic signature

To identify whether different clinical characteristics of LGG patients are associated with risk scores, we compared tumor histologic grade, gender, age, and IDH1 mutation status between high and low-risk groups in terms of risk scores. The LGG patients with WHO grade 3, age >40, IDH1-wildtype, unmethylated of MGMT promoter and 1p19q non-codel status had higher risk scores, but gender had no significant effect on risk scores (**Figures 3D–I**). To further clarify whether the model still has the ability to predict the prognosis of LGG patients among different subgroups, we performed further survival analyses in the above-mentioned subgroups. The high-risk group had a shorter OS, regardless of whether the LGG patients were older than 40 years old or younger (**Figures S1A, D**). Patients in the high-risk group also had a shorter OS between the different genders (**Figures S1B, E**). The 7-snoRNA prognostic signature also presented better predictive power for the prognosis of LGG patients between the high-risk and low-risk groups, in WHO grade 2 or WHO grade 3 subgroups (**Figures S1C, F**), and IDH mutant or wild type subgroups (**Figures S1G, H**). These results suggested that the 7-snoRNA prognostic signature is associated with the clinicopathological features of LGG patients and has good predictive ability for the prognosis of LGG patients in different subgroups.

### 3.5 Construction and validation of nomogram

**Figure 4:**
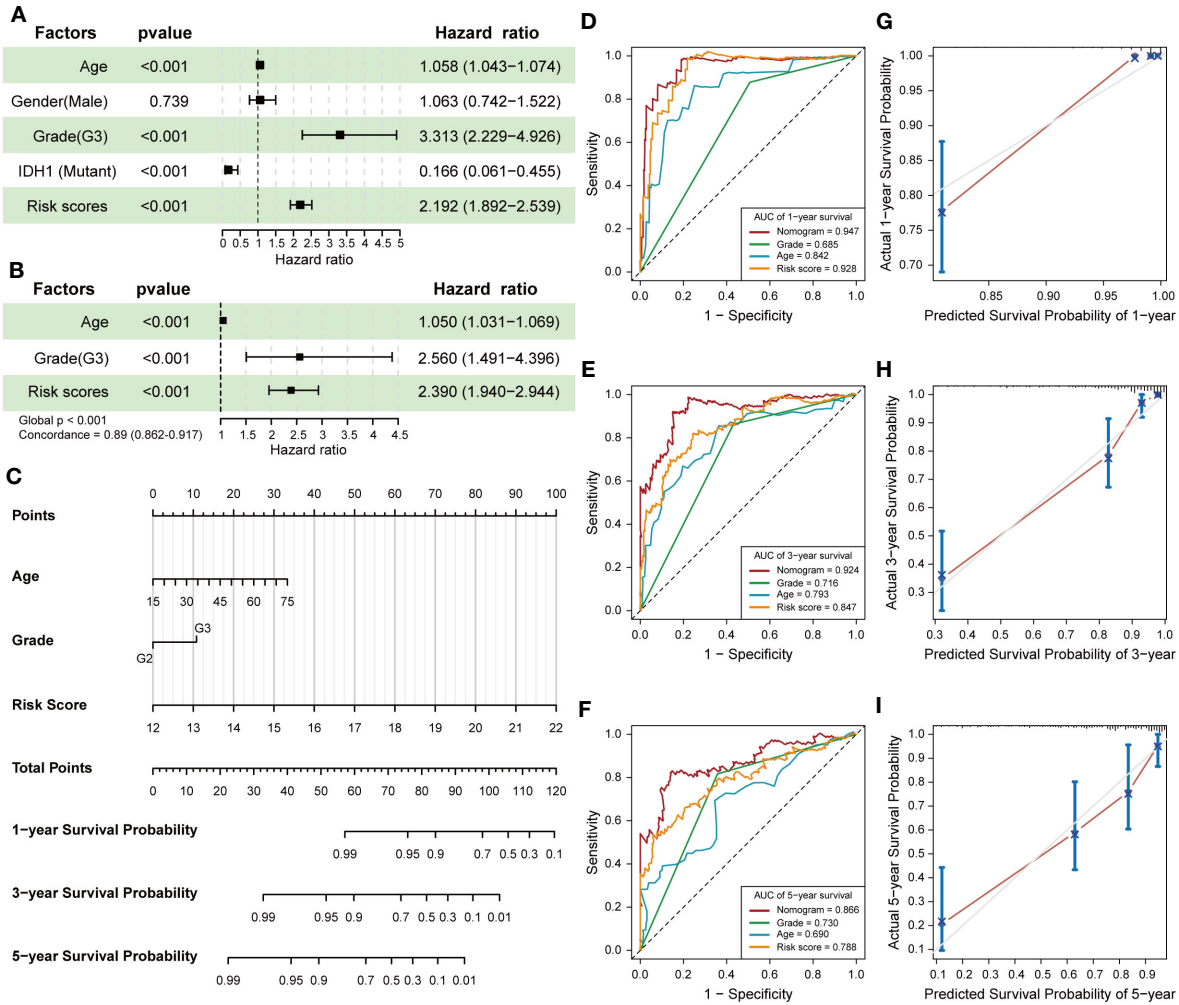
Establishment and validation of the Nomogram. **(A)** The forest plot revealed the univariable Cox regression results of age, gender, WHO grade, IDH1 mutation status and risk scores. **(B)** The results of the multivariate Cox proportional hazard regression model were constructed according to age, WHO grade and risk scores calculated by 7-snoRNA prognostic signature. **(C)** The nomogram visualizes the outcomes of the multivariate Cox proportional hazard regression model to predict the survival probability for LGG patients at 1-,3-,5-years. **(D-F)** Time-dependent ROC curves were plotted to illustrate the AUC of nomogram, WHO grade, age and risk score at 1-,3-,5-years in the training set. **(G-I)** Calibration plots showed the concordance between predicted survival probability and actual survival probability at 1-,3-,5-years in the training set.

The construction of a nomogram containing multiple LGG-related risk factors helps to more accurately predict the probability of survival in LGG patients. We first performed univariable Cox regression to analyze the effect of age, gender, WHO grade, IDH1 mutation status and risk score calculated by 7-snoRNA prognostic signature on the prognosis of LGG patients. The results revealed that old age, WHO grade 3, IDH1 wild type, higher risk scores were all risky factors (**Figure 4A**). Then we selected the age, WHO grade and risk scores to construct a multivariate Cox proportional hazard regression model, in which the global *P*-value< 0.001 and the concordance is 0.89 indicated that the predicted values were in good consistency with the actual results (**Figure 4B**). A nomogram was plotted to demonstrate the results of multivariable Cox regression of this prognostic model, and the 1-, 3-, 5-year survival probability of LGG patients was calculated by summing the scores of each independent risk factor (**Figure 4C**). To evaluate the predictive accuracy of the Nomogram, we plotted time-dependent ROC curves and calibration plots both in the training set and validation set. The AUC of Nomogram was higher than those of other risk factors in each ROC curves, suggesting that prediction of the survival probability of LGG patients based on the nomogram had a higher accuracy than that by using the 7-snoRNA prognostic signature alone both in the training set (**Figures 4D–F**) and validation set (**Figures S2A–C**). The calibration plots were drawn between the predicted survival probability and actual survival probability in 1, 3, 5 years, which also indicated that the prognostic model visualized by the nomogram had an excellent concordance both in the training set (**Figures 4G–I**) and validation set (**Figures S2D–F**).

### 3.6 Prediction of the potential biological functions of snoRNAs

**Figure 5:**
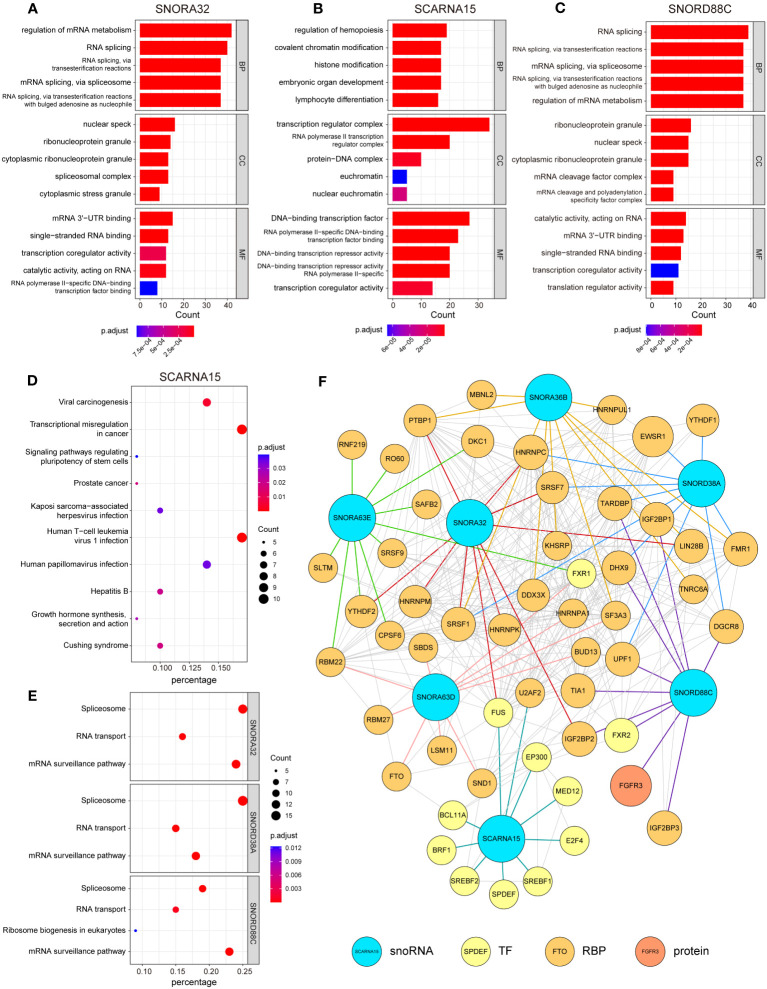
Analysis of RNA-protein interaction in relation to 7 snoRNAs. Enrichment analysis of 100 proteins that interact with each candidate snoRNAs in Gene Ontology (GO) enrichment **(A-C)** and Kyoto Encyclopedia of Genes and Genomes (KEGG) pathway enrichment analysis **(D, E)**. Each section shows the 5 most significant enrichment GO terms and 10 most significant KEGG pathways (SNORA32, SNORD38A and SNORD88C were only enriched in 3 or 4 pathways). Biological Process (BP), Cellular Component (CC), Molecular Function (MF). **(F)** A network drawn by the Cytoscape to illustrate the relationship between 7 candidate snoRNAs and its top 10 interacting proteins (TF, transcription factor; RBP, RNA-binding protein).

RNA-binding protein (RBP) is an important partner for RNA to perform its function, and is involved in various post-transcriptional regulation processes. RNAs interact with relevant RBPs to form functional units called ribonucleoprotein complexes, which are involved in the splicing, stabilization, translation, localization and degradation of RNA (25). RBPs exhibit aberrant regulation in many cancers, and disruption of the RBP-RNA interact network is causally linked to cancer development (26). To further explore the role played by these candidate snoRNAs in the development of glioma, we first screened the proteins that interacted with candidate snoRNAs in RNA Interactome Database (20). Next, we performed GO and KEGG enrichment analysis of the proteins that interacted with each snoRNA. The GO enrichment results showed that SNORA36B, SNORA32, SNORA63E, SNORA63D, SNORD38A and SNORD88C were mainly involved in biological processes (BP) such as RNA splicing and regulation of mRNA metabolism, while SCARNA15 was related to chromatin and histone modification and involved in embryonic development, hematopoiesis and other processes, which are not consistent with classical snoRNA. Cellular component (CC) was mainly enriched in nuclear speck, ribonucleoprotein granule, transcription regulator complex, etc. Molecular functions (MF) included binding to DNA, RNA and transcription factors (**Figures 5A–C**; **Figures S3A–D**). KEGG enrichment results revealed that SCARNA15, SNORA36B, SNORA63E and SNORA63D were related to viral carcinogenesis and transcriptional misregulation in cancer and involved in the infection process of some viruses, such as hepatitis B virus (HBV), human papillomavirus (HPV) and Human T-cell leukemia virus 1 (HTLV-1). In addition, SNORA36B, SNORA63E and SNORA63D were also involved in T-helper cell differentiation, and SNORA63E and SNORA63D participated in TNF signaling pathways and spliceosome (**Figure 5D**; **Figures S3E–G**). Moreover, SNORA32, SNORD38A and SNORD88C were mainly enriched in the spliceosome, RNA transport and mRNA surveillance pathway (**Figure 5E**). Finally, an interaction network showed the relationship between 7 candidate snoRNAs and its top 10 interacting proteins (**Figure 5F**).

Non-coding RNAs can also interact with other RNAs to form regulatory networks such as the competing endogenous RNA network, and changes in the expression of any component of the networks may disrupt the homeostasis of this complex system, ultimately leading to the development and progression of cancer (27). To map out the RNA interaction networks involved in these snoRNAs, we identified the RNAs that interacted with these snoRNAs through the database ENCORI (28) and RNA Interactome Database (20), queried the RNA interaction information of six snoRNAs and displayed them in a waffle chart.

**Figure 6:**
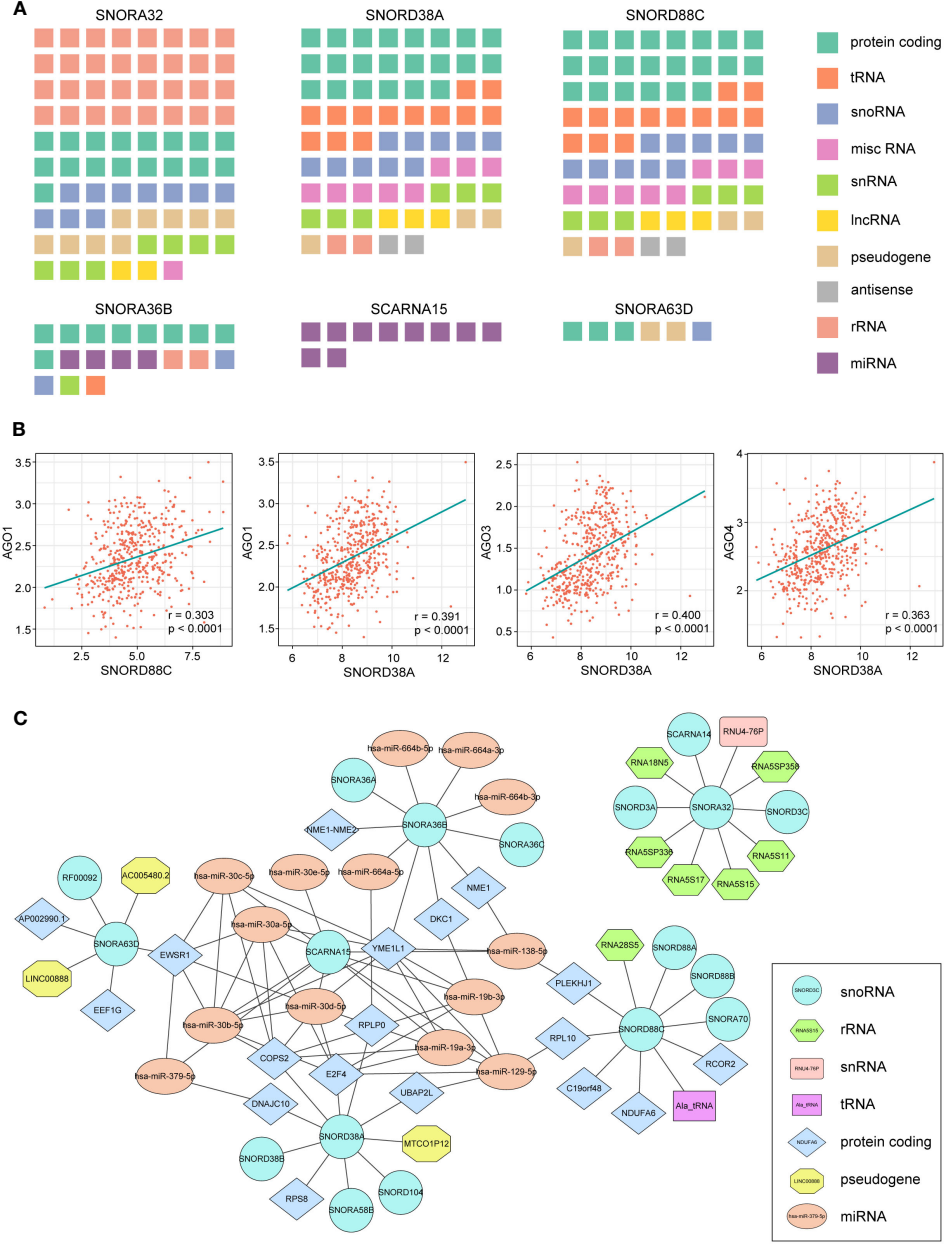
RNA-RNA interaction analysis of 7 snoRNAs. **(A)** Waffle charts illustrate the RNAs predicted by the ENCORI and RNA Interactome Database which can interact with snoRNAs (misc RNA, miscellaneous RNA; snRNA, small nuclear RNA). **(B)** The expression of SNORD88C and SNORD38A showed a significant association with AGO proteins. **(C)** The network of six snoRNAs and their top 10 interaction RNAs.

There were more RNAs interacting with the SNORA32, SNORD38A and SNORD88C than other snoRNAs, which involved many types of RNA, among which SNORA32 mainly interacted with various rRNAs and mRNAs, while SNORD38A and SNORD88C mainly interacted with mRNAs and tRNAs (**Figure 6A**). SCARNA15, unlike others, mainly interacted with miRNAs, suggesting that SCARNA15 has a different function from classical snoRNAs. Christine Ender et al. confirmed experimentally that SCARNA15 is able to bind to Argonaute (Ago) proteins and exerts miRNA-like effects (29). This kind of snoRNAs was named as Dual function sno-miRNAs, which means they have the dual functions of classical snoRNA and miRNA (30). To further investigate whether the remaining snoRNAs may also have miRNA-like effects, we calculated the correlation coefficient between the expression of snoRNAs and Ago proteins. We set the correlation coefficient greater than 0.3 and *P*< 0.05 as a significant correlation, and the results showed that SNORD38A and SNORD88C were associated with Ago proteins, which led to the speculation that these two snoRNAs might bind to AGO proteins and play miRNA-like roles (**Figure 6B**). Finally, we selected the 10 RNAs with the highest interaction scores with each snoRNAs to construct the snoRNA-RNA interaction network (**Figure 6C**).

To further investigate whether there are differences in gene sets between the high-risk and low-risk groups, we performed a Gene Set Enrichment Analysis (GSEA). GSEA results revealed that there were 7 hallmarks significantly enriched in the high-risk group, including apical junction, IL-2-STAT5 signaling, glycolysis, reactive oxygen species pathway, estrogen response late, PI3K-AKT-mTOR signaling, and peroxisome (**Figure S4**).

### 3.7 Immune landscape of the different subgroups based on 7-snoRNA model

**Figure 7:**
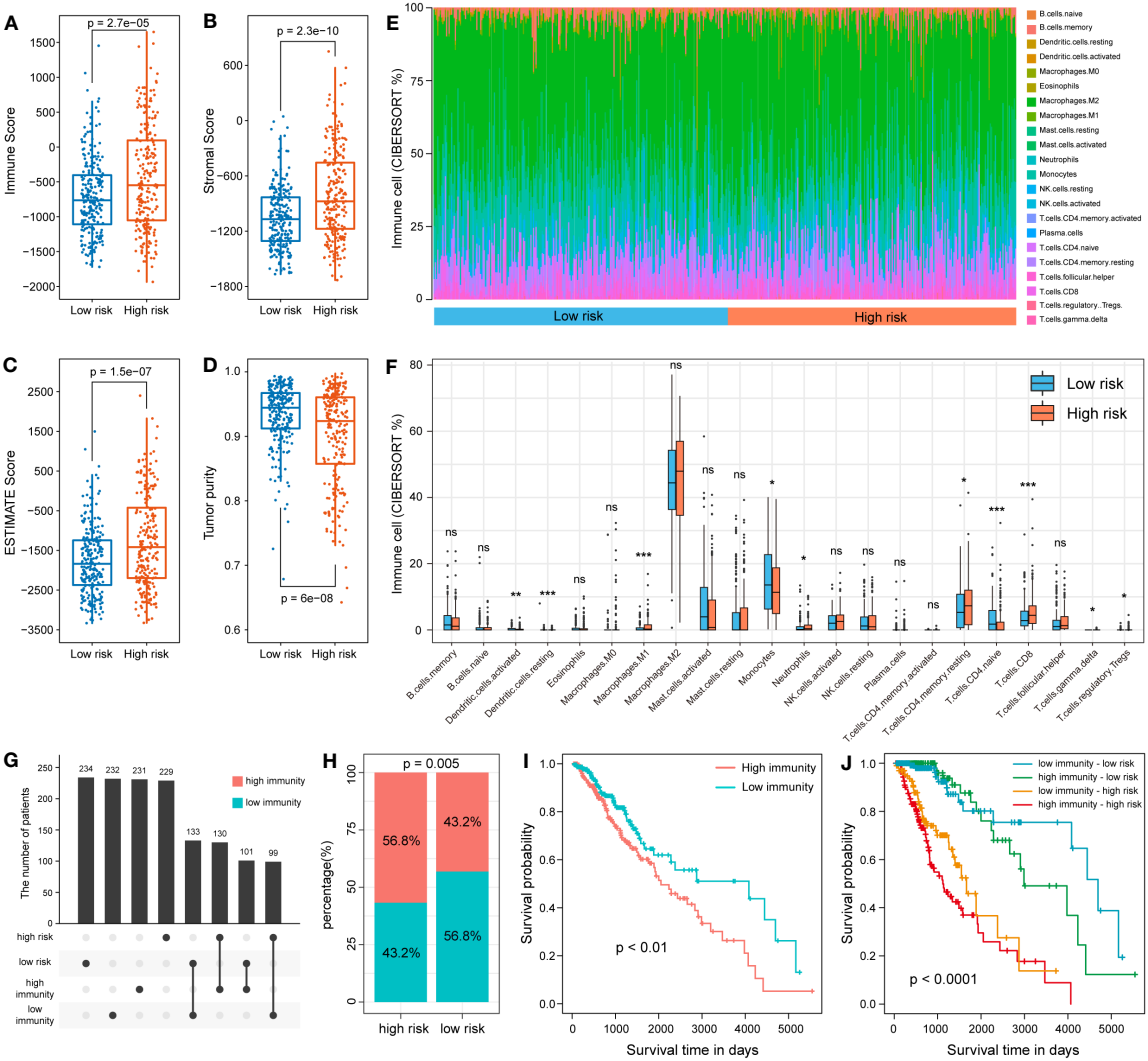
Analysis of 7-snoRNA models and tumor immune microenvironment. The Immune Score **(A)**, Stromal Score **(B)**, ESTIMATE Score **(C)** and Tumor purity **(D)** in different subgroups. **(E, F)** Comparison of the abundance of 22 types of immune cells in different risk groups. **(G, H)** Distribution of patients in the different immune and risk groups. **(I)** Survival analysis of the high- and low- immunity group. **(J)** Survival analysis of the interaction between the two groups.

Previous enrichment analysis revealed that these snoRNAs were associated with the immune response, so we further explored the relationship between the 7-snoRNA model and the tumor immune microenvironment. After calculating the scores for the low- and high- risk groups by the ESTIMATE algorithm, it was found that the ESTIMATE Score, Immune Score and Stromal Score were higher in the high-risk group than in the low-risk group and the tumor purity scores were lower (**Figures 7A–D**). In addition, the abundance of 22 different immune cells was calculated for each patient using the CIBERSORT algorithm. The results are shown in **Figures 7E, F**, where M1 macrophages and CD8**^+^** T cells were at higher levels in the high-risk group while CD4**^+^** naïve T cells and dendritic cells were at higher levels in the low-risk group. To further explore the interaction of risk grouping and immune score on prognostic impact, we divided the patients into two groups based on ESTIMATE score and found a higher percentage of high-immunity group in the high-risk group (**Figures 7G, H**). Survival analysis revealed that patients in the high-immunity group had a worse prognosis, and interaction analysis between the different groups showed that patients in the high-immunity and high-risk groups had the worst prognosis, while patients in the low-immunity and low-risk groups had the best prognosis (**Figures 7I, J**).

### 3.8 Immune-related gene expression profiles of 7-snoRNA model

**Figure 8:**
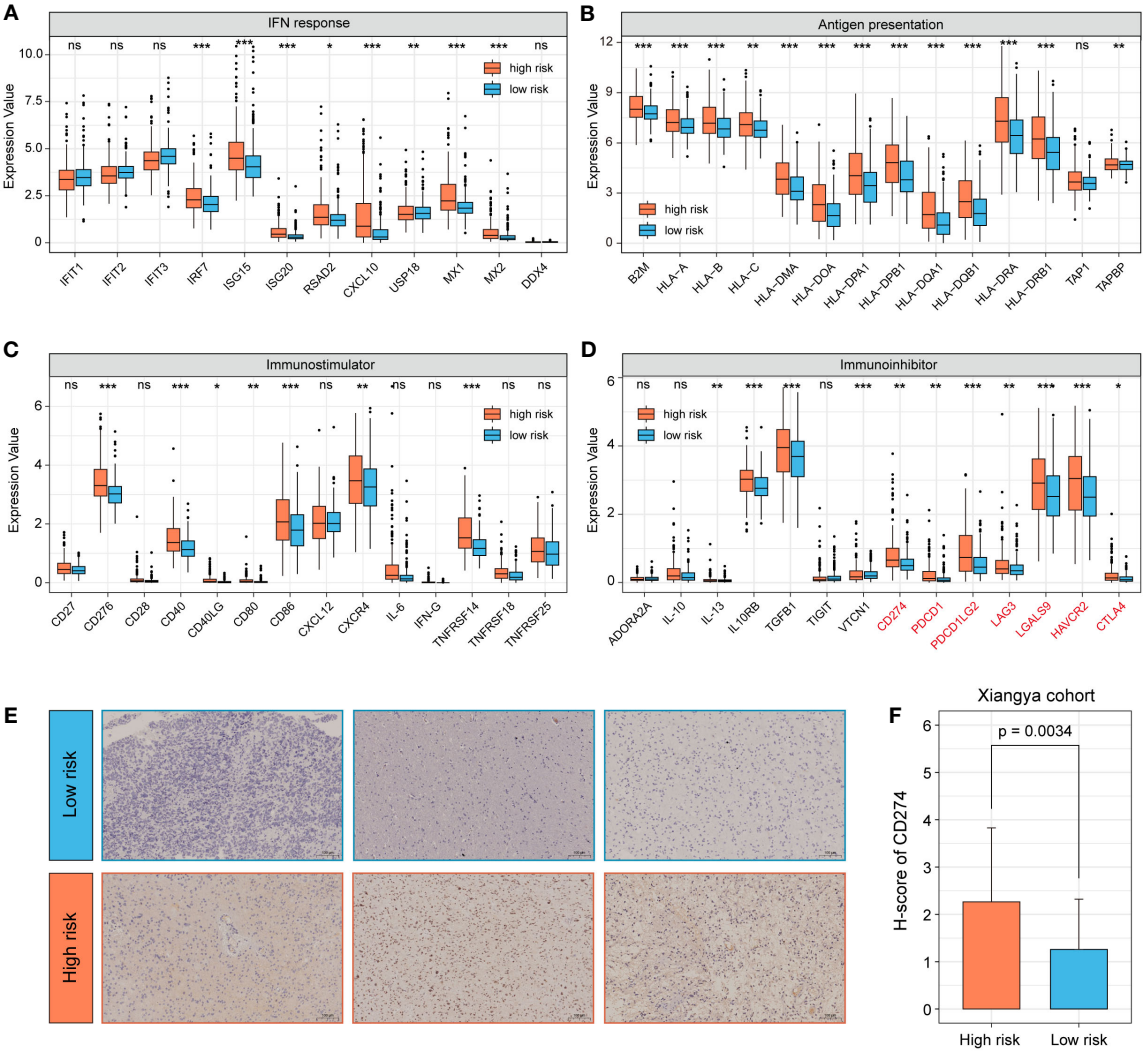
Analysis of immune-related gene expression profiles of 7-snoRNA model. The expression of IFN response related genes **(A)**, antigen presentation genes **(B)**, stimulatory immune-related genes **(C)** and immunosuppressors (Immune checkpoint molecules were marked in red) **(D)** in different subgroups. Representative immunohistochemical images of CD274 (200×) for different subgroups of clinical samples **(E)** and statistical results of H-score **(F)**. *P< 0.05, **P< 0.01 and ***P< 0.001, ns means ‘no significance’.

The expression of immune-related genes can reflect the immune status of different patients and whether they can benefit from immunotherapy. We compared the expression of different subgroups in a multiple immune-related gene set. The results indicated that the high-risk group had higher expression levels in IFN response and antigen presentation related genes (**Figures 8A, B**). In addition, we noticed that the some immunostimulatory molecules, such as CD276, CD40 and TNFRSF14 showed higher expression levels in the high-risk group (**Figure 8C**). Among immunosuppressive molecules, expression of multiple immune checkpoints molecules, such as CD274, PDCD1 and LAG3 was significantly higher in the high-risk group (**Figure 8D**). As an important immune checkpoint molecule, the expression of CD274 is of guiding significance for immunotherapy of tumors. Therefore, we further validated it by clinical samples. 82 glioma specimens collected by the Department of Neurosurgery of Xiangya Hospital were made into tissue microarrays and performed immunohistochemical experiments. Additional qRT-PCR was performed to detect the 7 snoRNAs expression of each sample to calculate the risk score. The results demonstrated that the H-score of CD274 was higher in the high-risk group, which was consistent with the results of our bioinformatics analysis (**Figures 8E, F**).

### 3.9 SNORD88C promotes the proliferation, invasion and migration of glioma cells

**Figure 9:**
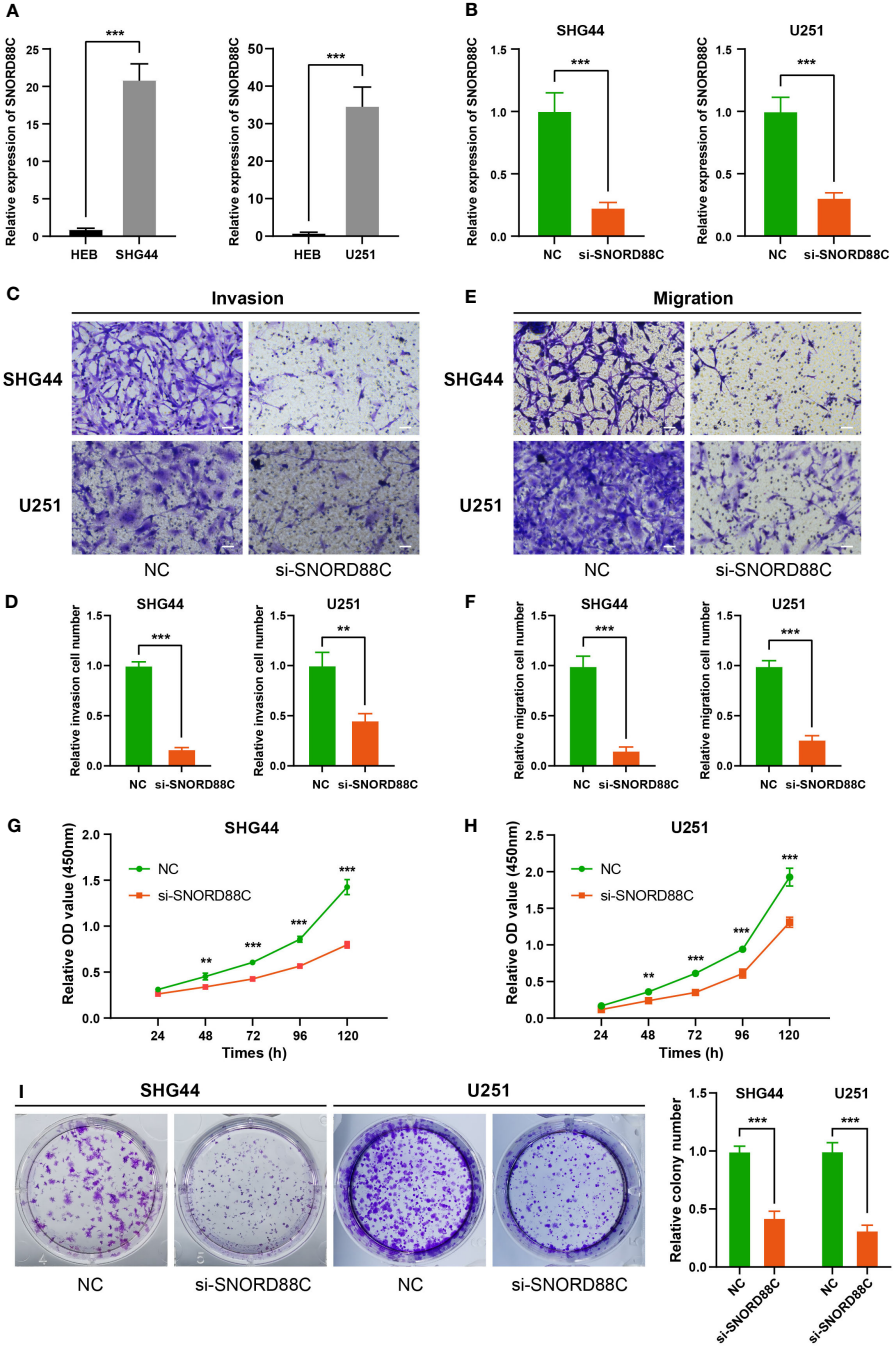
Knockdown of SNORD88C attenuates the invasion, migration and proliferation of glioma cells. **(A)** The relative expression of SNORD88C was measured by qRT-PCR in normal glial cell line HEB and glioma cell lines SHG44 and U251. **(B)** The transfection efficiency of siRNA in SHG44 and U251 cells after transfection with si-SNORD88C (siRNA targeting SNORD88C) was quantified by qRT-PCR. **(C, D)** The invasion ability of SHG44 and U251 cells were measured by Transwell assay, scale bars = 50μm. **(E, F)** The migration ability of SHG44 and U251 cells in both groups was determined by the Transwell assay (no Matrigel), scale bars = 50μm. **(G, H)** The proliferation of SHG44 and U251 cells with or without SNORD88C knockdown was measured every 24 hours by the CCK-8 assay. **(I)** Colony formation assay was performed to detect the difference in proliferative capacity and viability of SHG44 and U251 cells between the two groups. Data are expressed as the mean values ± SD. **P< 0.01 and ***P< 0.001.

**Figure 10:**
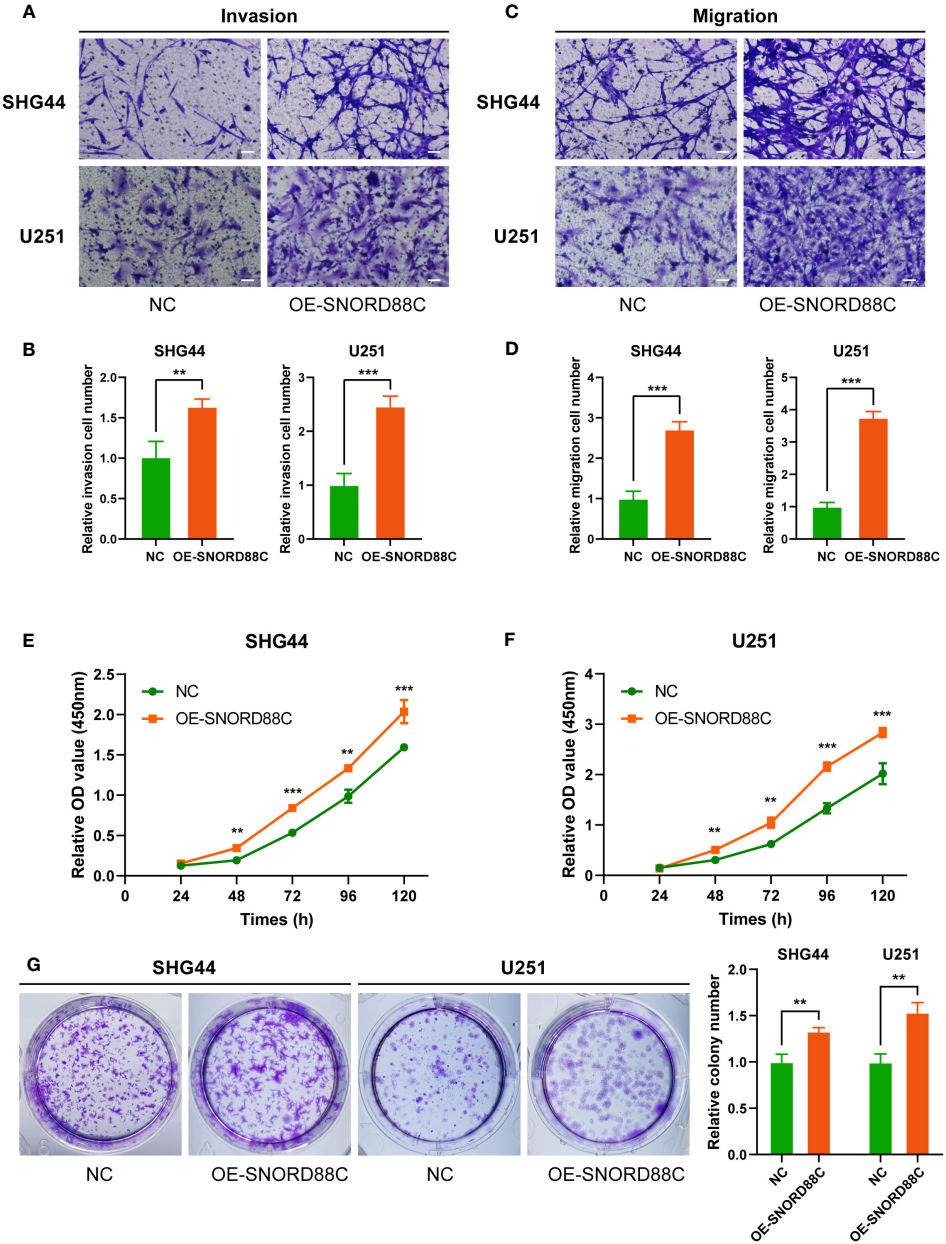
Overexpression of SNORD88C enhances the invasion, migration and proliferation of glioma cells. **(A, B)** The invasion ability of SHG44 and U251 cells in two groups were measured by Transwell assay, scale bars = 50μm. **(C, D)** The migration ability of SHG44 and U251 cells in both groups was determined by the Transwell assay (no Matrigel), scale bars = 50μm. **(E, F)** The proliferation of SHG44 and U251 cells with or without overexpression of SNORD88C was measured every 24 hours by the CCK-8 assay. **(G)** Colony formation assay was performed to detect the difference in proliferative capacity and viability of SHG44 and U251 cells between the two groups. Data are expressed as the mean values ± SD. **P< 0.01 and ***P< 0.001.

Based on the results of above bioinformatics analysis, we supposed that SNORD88C plays an important role in the development of glioma, and therefore we performed *in vitro* experiments to validate it. The qRT-PCR results showed that the expression of SNORD88C was significantly higher in the glioma cell lines (SHG44 and U251) than in the normal glial cell line HEB (**Figure 9A**). Small interfering (siRNA) was applied to knockdown the expression of SNORD88C to investigate its biological function in glioma. After transfection of SHG44 with si-SNORD88C (siRNA targeting SNORD88C), there was a significant decrease in the expression level of SNORD88C in the glioma cells, which indicated that the interference was effective (**Figure 9B**). Migration and invasive abilities are important biological phenotypes of glioma cells. The results of Transwell assay showed that knockdown of SNORD88C expression significantly decreased the invasion ability and migration ability of glioma cells (**Figures 9C–F**). As indicated by CCK-8 assay, the cell proliferation ability was significantly inhibited in the si-SNORD88C group compared to the negative control (NC) group (**Figures 9G–H**). Finally, the outcomes of the colony formation assay revealed that SHG44 and U251 cells with knockdown of SNORD88C were significantly decreased in the count and volume of colonies as compared to the NC group (**Figure 9I**). In addition, we examined the effects on glioma cells after overexpression of SNORD88C. Transwell assay showed that overexpression of SNORD88C increased the invasive and migratory capacity of glioma cells (**Figures 10A–D**). CCK-8 assay and clone formation assay revealed that overexpression of SNORD88C promotes glioma cell proliferation (**Figures 10E–G**). These findings suggest that SNORD88C promotes glioma cell proliferation, migration and invasion.

### 3.10 Potential mechanisms for SNORD88C to perform biological functions

**Figure 11:**
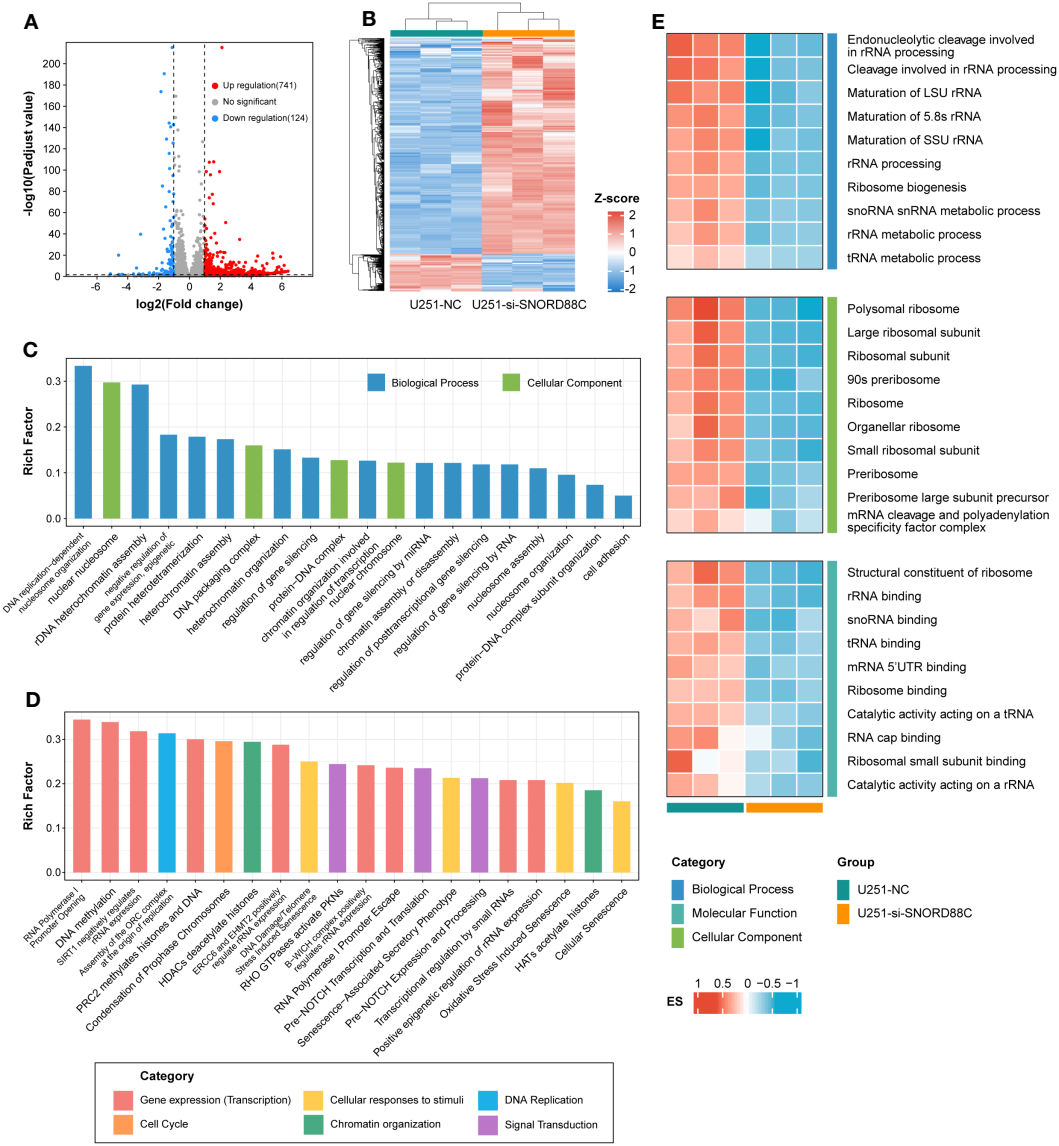
RNA-seq reveals the mechanism by which SNORD88C exerts biological functions in glioma cells. **(A)** The volcano plot illustrates the distribution of differentially expressed genes. **(B)** The clustering heatmap demonstrates the expression of the differential genes between the two groups. Differentially expressed genes were analyzed for GO enrichment analysis **(C)** and Reactome enrichment analysis **(D)**, and the top 20 terms with the most significant enrichment are shown. **(E)** Heatmap demonstrating GSVA results for RNA-related pathways between the two groups.

To further explore the potential mechanisms by which SNORD88C exerts biological functions in glioma cells, we performed RNA-seq after knockdown of SNORD88C expression in U251 cells. A total of 865 differentially expressed genes were identified after differential gene expression analysis of the RNA-seq results (**Figures 11A, B**). These differentially expressed genes were further analyzed for GO enrichment analysis and Reactome enrichment analysis, and the 20 terms with the most significant enrichment are displayed respectively (**Figures 11C, D**). Enrichment analysis showed that SNORD88C is involved in biological processes such as DNA replication, DNA damage repair, chromatin assembly, RNA transcription process and regulation of gene expression level. Since the analysis results from public databases indicated that SNORD88C was closely related to RNA metabolism and catabolism, we further performed GSVA analysis targeting RNA-related pathways in the RNA-seq results (**Figure 11E**). The results demonstrated that knockdown of SNORD88C exhibited a significant decrease in the activity of pathways associated with rRNA, tRNA and mRNA, suggesting that SNORD88C is closely related to RNA metabolism and exerts a biological function in gliomas through the regulation of RNA levels.

## 4 Discussion

SnoRNAs are a group of non-coding RNAs with 60-300 nucleotides in length and are mainly located in the nucleolus. It is generally believed that snoRNAs are mainly involved in post-transcriptional modifications of ribosomes, snRNAs and other target molecules (10, 11). However, in recent years, an increasing number of studies have found a closer relationship between snoRNA and the progression of cancer, such as SNORD42A in leukemia cells (13), SNORA65 and SNORA7A/7B in lung cancer cells (14), and SNORD44 in glioma cells (15). In addition, some researchers have found that several snoRNAs can act as prognostic markers in cancer and have the effect of evaluating the prognosis of tumor patients (16, 17).

Glioma is the most prevalent central nervous system tumor with high malignancy and poor prognosis, and the discovery of new prognostic molecules is needed to evaluate the prognosis of patients. Therefore, we constructed a 7-snoRNA prognostic signature for predicting it. Firstly, we screened 122 snoRNAs from the TCGA database and SNORic database that were associated with survival of glioma patients. Secondly, by using LASSO regression and multivariate Cox regression, we selected the 7 most critical snoRNAs from 122 survival-related snoRNAs and constructed a prognostic signature. Finally, we combined the 7-snoRNA prognostic signature and the relevant clinical characteristics of glioma patients to construct a nomogram that could be utilized to better predict the prognosis of glioma patients. The ROC curves revealed that the 7-snoRNA prognostic signature and the nomogram had high sensitivity and specificity in both the training and validation sets.

Among the screened 7 snoRNAs, SNORA32, SNORA36B, SCARNA15, SNORD88C, and SNORD38A were protective factors, while SNORA63D and SNORA63E were risk factors. Except SCARNA15, the remaining snoRNAs have not been studied by other researchers. In order to investigate the role they play in glioma, we performed functional prediction analyses. It is generally believed that snoRNA binds to proteins, forms small nucleolar ribonucleoproteins (snoRNPs), and participates in the modification of rRNA as well as the assembly of ribosomes (31). Therefore, we first searched the database for proteins that can interact with each snoRNA, which are mainly RBPs and transcription factors. Subsequently, we performed GO and KEGG enrichment analysis of these interacting proteins to speculate on the potential biological functions of each snoRNA. GO and KEGG enrichment results revealed that most of these proteins are enriched to perform post-transcriptional regulatory roles such as RNA splicing, metabolism and modifications, suggesting that these snoRNAs can bind to RBPs and regulate the post-transcriptional modification process of multiple RNAs. Consistent with our findings, Dong et al. found that the MSI2 (a kind of RBP) can enhance the stability of SNORD12B to regulate post-transcriptional modifications of ZBTB4 mRNA and affect glycolipid metabolism in glioblastoma cells (32).

In addition to the snoRNA-protein interaction, we further analyzed the relationship between snoRNAs and other RNAs. Research has found that snoRNA can be degraded into shorter RNA fragments that exert biological functions different from the snoRNA itself, called sdRNA (sno-derived RNAs) (33). Patterson et al. have demonstrated that SNORNA93-derived sdRNA93 has microRNA-like effects and promotes invasion of breast cancer cells by reducing pipox expression (34). U3-derived miR-U3 can work like microRNA *in vivo*, targeting the 3’UTR of sortin nexin 27 mRNA (35). Our results showed that SNORA32, SNORD38A and SNORD88C interacted with multiple mRNAs, further analysis of the correlation between AGO proteins and snoRNAs revealed that SNORD88C and SNORD38A were correlated with AGO protein expression, thus it was hypothesized that these two snoRNAs may also produce sdRNAs and fulfill microRNA-like roles.

Our study found a significant increase in the degree of immune infiltration in the high-risk group, with elevated levels of multiple immune cells, including CD8**^+^** T cells. Intratumoural CD8**^+^** T cells are affected by the tumor immune microenvironment and no longer exert the ability to eliminate cancers, becoming dysfunctional CD8**^+^** T cell population (36). In addition, dysfunctional CD8**^+^** T cell population is significantly different from classical CD8**^+^** T cell in function, and may exhibit decreased secretion of cytokines such as tumour necrosis factor (TNF) and interferon- γ (IFNγ), as well as increased expression of inhibitory receptor genes such as PDCD1, LAG3 and CTLA4 (37). Our results suggested that PDCD1, LAG3, CTLA4 expression levels were increased in the high-risk group while IFNγ levels were not different from the low-risk group. This also indicates from another aspect that the increase of CD8**^+^** T cells in the high-risk group may be predominantly dysfunctional CD8**^+^** T cells.

CD274, an important immunotherapeutic target, has achieved good results in non-small cell lung cancer, colorectal cancer and melanoma, and has also demonstrated prolonged overall survival in several phase II clinical trials in glioma (38). We found elevated levels of CD274 expression in the high-risk group by bioinformatics analysis, which was further validated in clinical samples by immunohistochemical assays. This suggests that 7-snoRNA prognostic signature may be able to predict how well patients will respond to immunotherapy and that patients in the high-risk group may have a better clinical benefit from PD-L1 blocker therapy.

From the results of bioinformatics analysis indicated that SNORD88C has a major role in the development of glioma, and then *in vitro* experiments were performed to validate it. The expression of SNORD88C was significantly higher in the glioma cell lines SHG44 and U251 than in the normal astrocyte line HEB. SNORD88C could promote the proliferation, migration and invasion of glioma cells. RNA-seq results indicate that SNORD88C is involved in DNA replication, DNA methylation, DNA damage repair and regulation of gene expression levels in gliomas, and especially has a significant effect on RNA-related pathways. These results suggest that SNORD88C exerts a promotional effect on glioma cells by participating in multiple biological processes and may serve as a potential therapeutic target for glioma.

Although we constructed a 7-snoRNA prognostic signature for predicting glioma prognosis and performed functional analysis, there is still much room for improvement in our research. Since there are very few sequencing datasets about snoRNA in glioma and only sequencing data for TCGA-LGG cohort are available in SNORic database. This led us to construct a prognostic signature for LGG only, not for the whole glioma. In addition, our assessment of the predictive performance of the constructed prognostic signature was somewhat biased due to the lack of other datasets as external validation sets. In addition, bioinformatics analysis suggested that SNORD88C functions as an oncogenic gene in glioma, which was validated by cellular phenotype experiments. The specific underlying mechanism in more detail remains to be investigated in our future work.

## 5 Conclusion

This study reveals that snoRNA has an impact on the prognosis of LGG patients and can be used as a potential prognostic marker. We established a 7-snoRNA prognostic signature and nomogram that can be applied to evaluate the survival of LGG patients with good sensitivity and specificity. Further exploration of the functions for candidate snoRNAs revealed that SNORD88C could promote the proliferation, migration and invasion of glioma cells and is involved in a variety of biological processes related to DNA and RNA.

## Data availability statement

The original contributions presented in the study are included in the article/**Supplementary Material**. Further inquiries can be directed to the corresponding authors.

## Ethics statement

The studies involving humans were approved by Ethic Committee of the Xiangya Hospital of Central South University. The studies were conducted in accordance with the local legislation and institutional requirements. The participants provided their written informed consent to participate in this study.

## Author contributions

Conceived and designed this study: XJ, WL and CR. Wrote the manuscript: YZ. Data collection, analysis, and visualization: YZ, WY, YK, ZW and HH. All authors contributed to the article and approved the submitted version.
