# Supplementary material for: A prognostic signature based on snoRNA predicts the overall survival of lower-grade glioma patients
Source: Front Immunol. 2023 Nov 1;14:1138363. doi: 10.3389/fimmu.2023.1138363 (PMC10646524; doi:10.3389/fimmu.2023.1138363)
Supplement: Supplementary file 1 [file DataSheet_1.docx]

Supplementary Material

Yi Zhou^1,2^, Wen Yin^1,2^, Yirui Kuang^1,2^, Zhaoping Wu^1,2^, Haoxuan Huang^1,2^, Weidong Liu^1,2,3,4*^, Xingjun Jiang^1,2*^, Caiping Ren^1,2,3,4*^

* Corresponding should be addressed to:

Xingjun Jiang, Email: jiangxj@csu.edu.cn

Caiping Ren, Email: rencaiping@csu.edu.cn


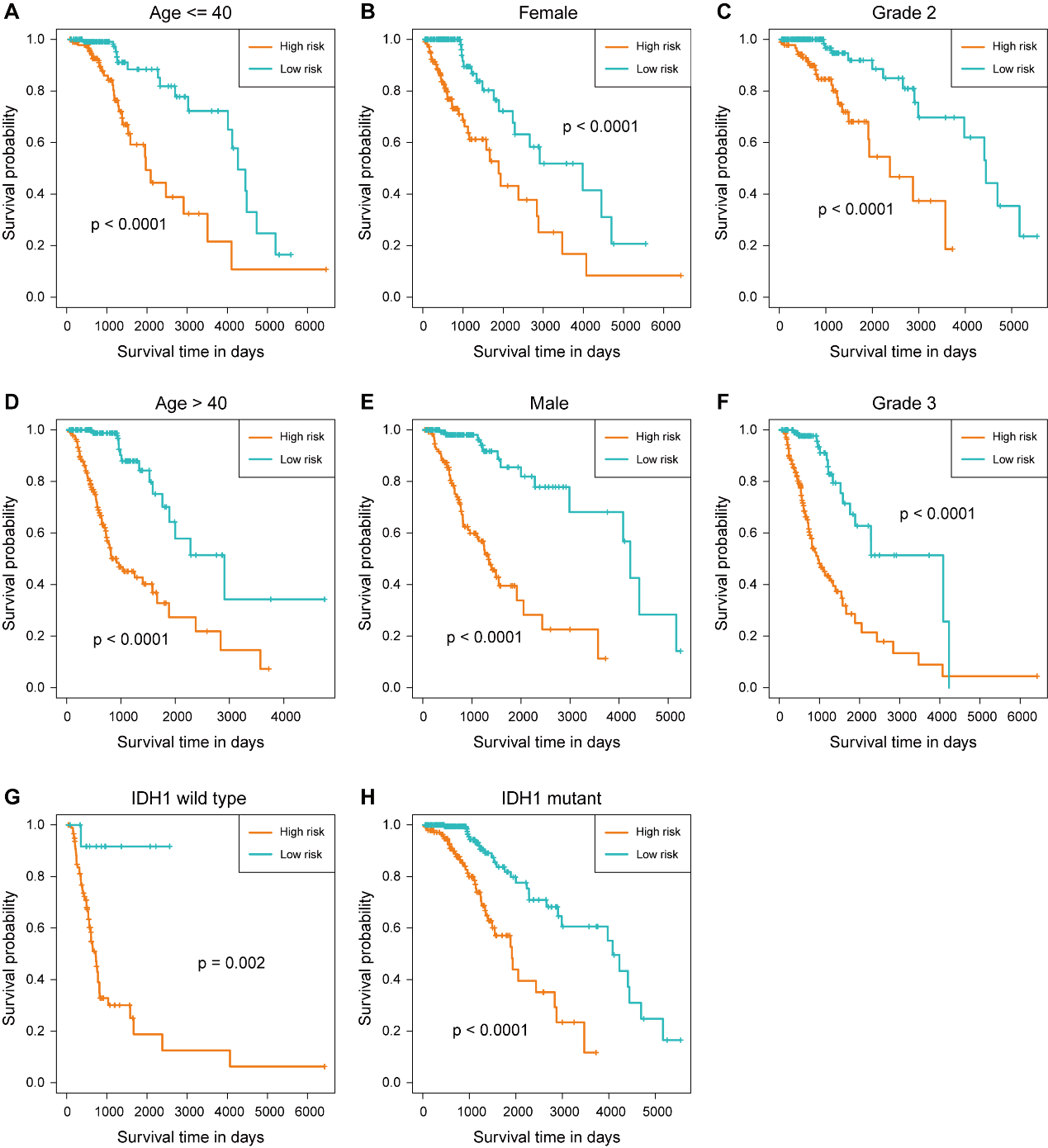


**Figure S1: Stratification analysis of 7-snoRNA prognostic signature.** Kaplan–Meier survival curve was plotted to compare the OS between the high-risk group and low-risk group in different LGG patient subgroups divided according to different clinicopathological features.


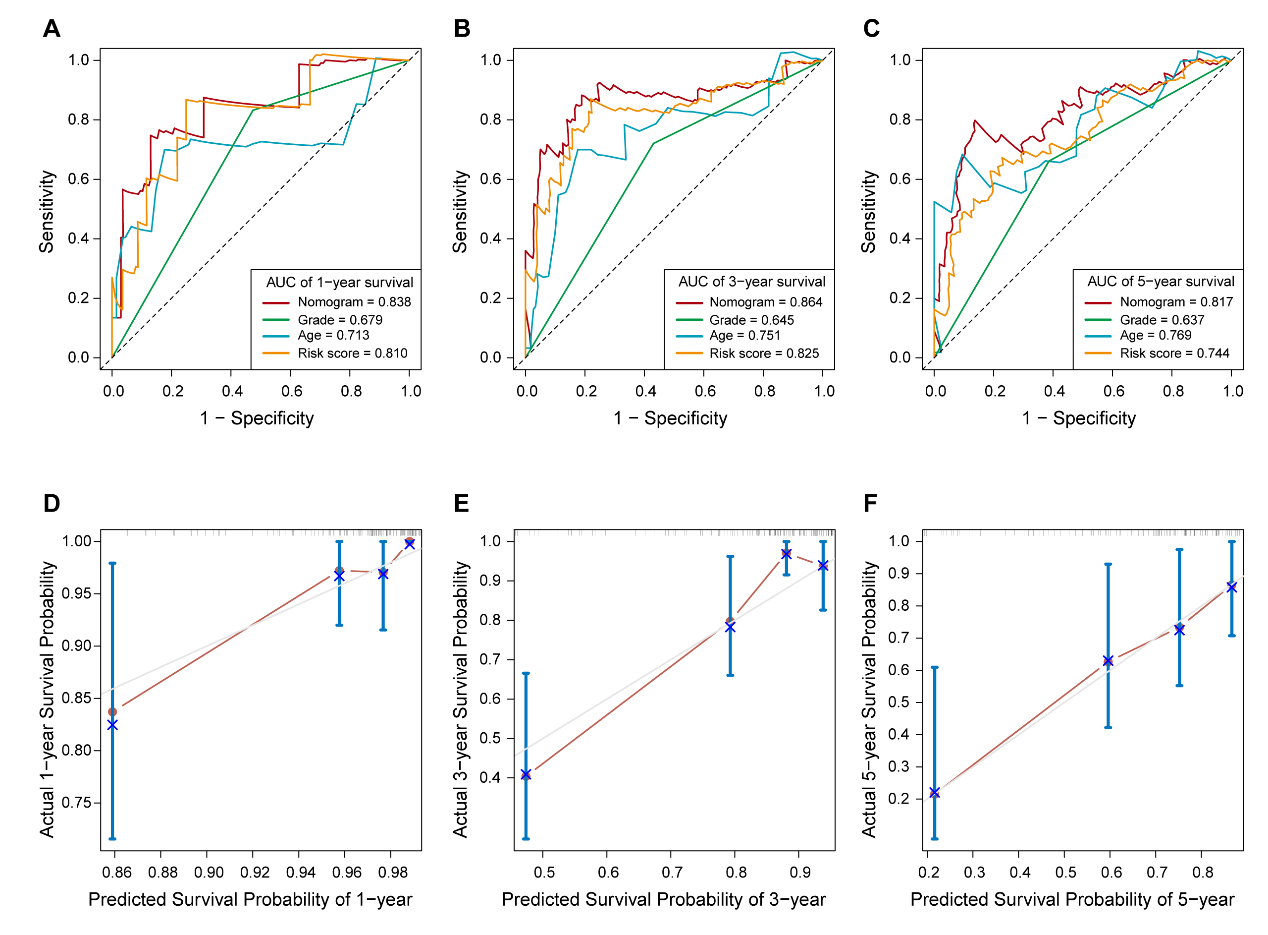


**Figure S2: Validation of the nomogram.** Time-dependent ROC curves were plotted to illustrate the AUC of nomogram, WHO grade, age and risk score at 1-,3-,5-years in the validation set. Calibration plots showed the concordance between predicted survival probability and actual survival probability at 1-,3-,5-years in the validation set.


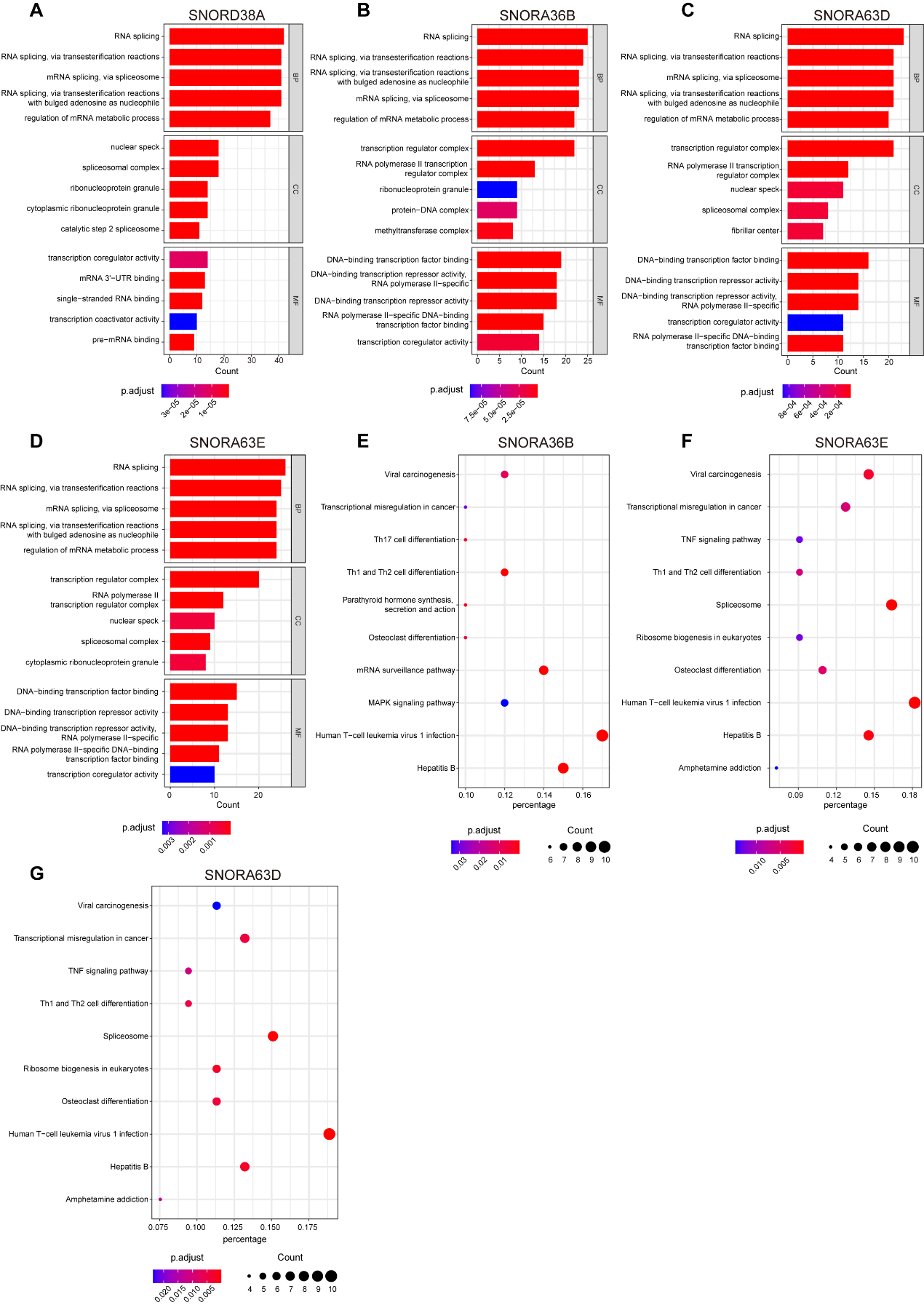


**Figure S3:** Enrichment analysis of 100 proteins that interact with each candidate snoRNAs in Gene Ontology (GO) enrichment and Kyoto Encyclopedia of Genes and Genomes (KEGG) pathway enrichment analysis. Each section shows the 5 most significant enrichment GO terms and the 10 most significant KEGG pathways.


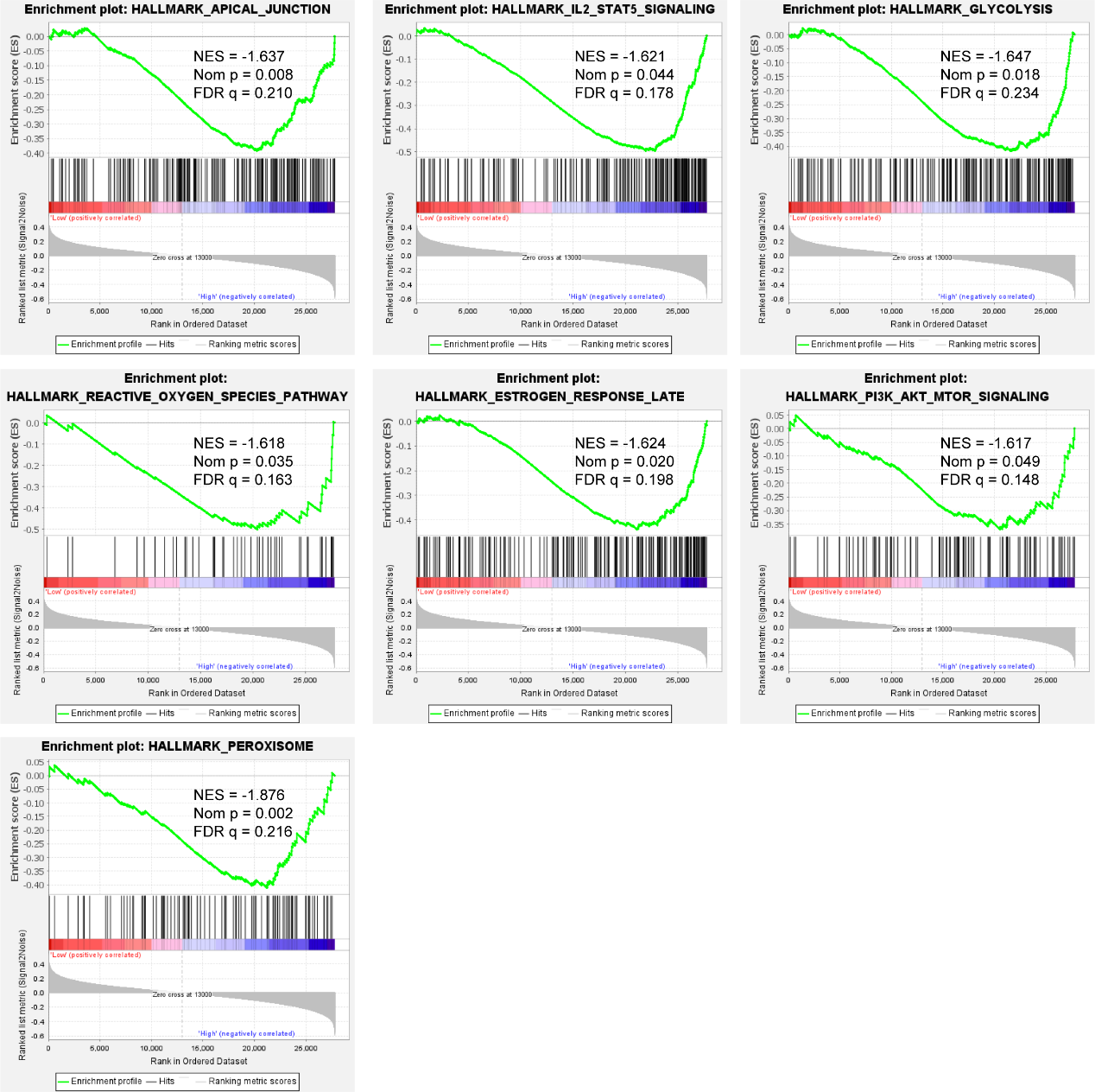


**Figure S4:** Gene Set Enrichment Analysis (GSEA) revealed the 7 most significant enriched hallmark gene sets.


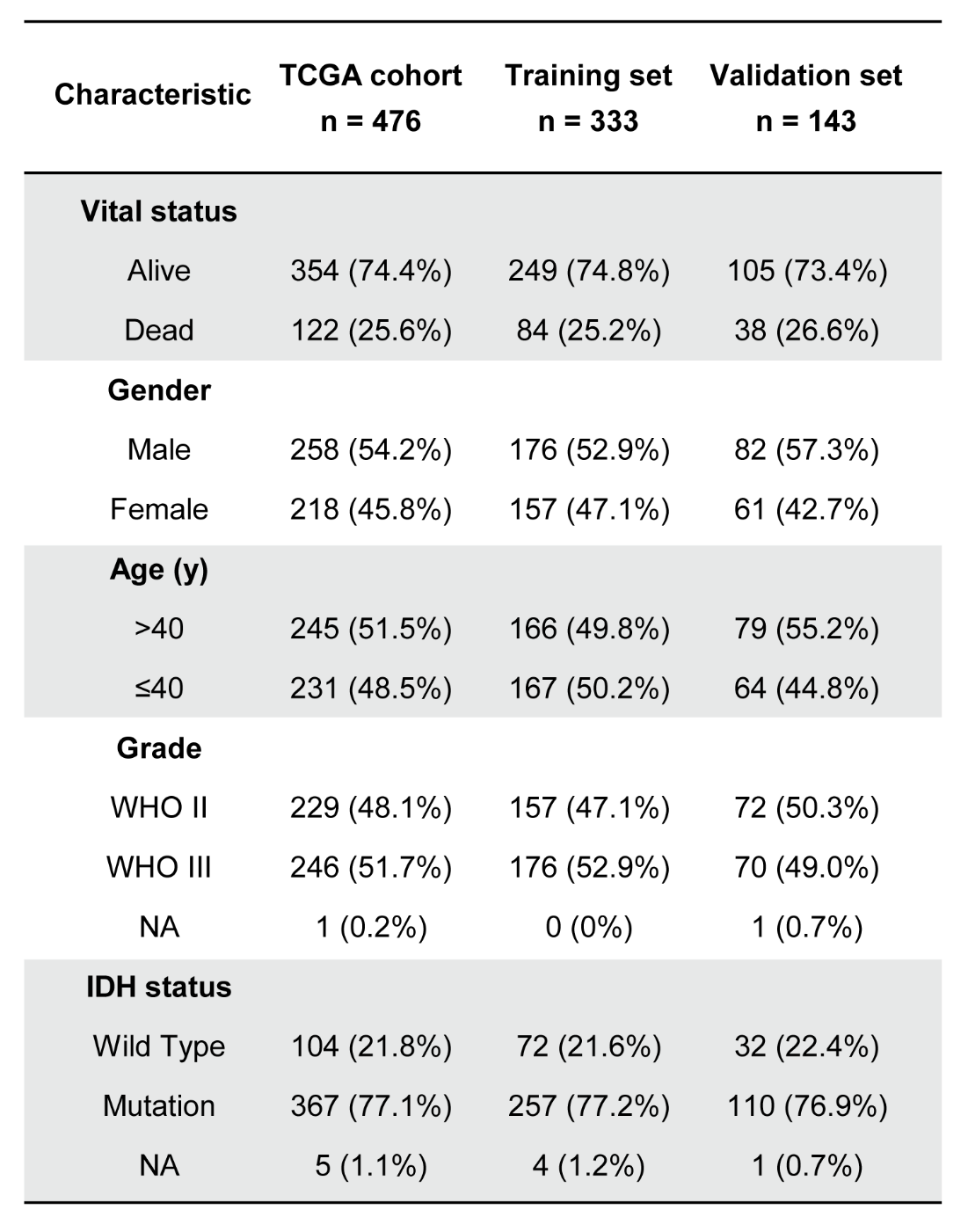


**Table S1:** Clinical characteristics of 476 patients in the LGG cohort from TCGA in this study.
